# Proteomic Changes Associated with Successive Reproductive Periods in Male Polychaetous *Neanthes arenaceodentata*

**DOI:** 10.1038/srep13561

**Published:** 2015-09-04

**Authors:** Kondethimmanahalli H. Chandramouli, Donald Reish, Huoming Zhang, Pei-Yuan Qian, Timothy Ravasi

**Affiliations:** 1 KAUST Environmental Epigenetic Program (KEEP), Division of Biological and Environmental Sciences & Engineering, Division of Applied Mathematics and Computer Sciences. King Abdullah University of Science and Technology, Kingdom of Saudi Arabia; 2 Department of Biological Sciences, California State University, Long Beach, California, United States; 3 Bioscience Core Laboratory, King Abdullah University of Science and Technology, Thuwal, Kingdom of Saudi Arabia; 4 KAUST Global Collaborative Research Program, Division of Life Science, The Hong Kong University of Science and Technology, Hong Kong.

## Abstract

The polychaetous annelid *Neanthes acuminata* complex has a widespread distribution, with the California population referred to as *N. arenaceodentata*. The reproductive pattern in this complex is unique, in that the female reproduces once and then dies, whereas the male can reproduce up to nine times. The male incubates the embryos until the larvae leave the male’s tube 21–28 days later and commences feeding. Reproductive success and protein expression patterns were measured over the nine reproductive periods. The percent success of the male in producing juveniles increased during the first three reproductive periods and then decreased, but the number of juveniles produced was similar through all nine periods. iTRAQ based quantitative proteomics were used to analyze the dynamics of protein expression patterns. The expression patterns of several proteins were found to be altered. The abundant expression of muscular and contractile proteins may have affected body weight and reproductive success. Sperm have never been observed; fertilization occurs within the parent’s tube. Proteins associated with sperm maturation and fertilization were identified, including ATPase, clathrin, peroxiredoxins and enolase, which may provide clues to the molecular mechanisms enabling males to reproduce multiple times.

The polychaetous annelid species complex of *Neanthes acuminata* has a widespread distribution, with names depending on geographic location. For example, the species present in southern California, Baja California, Hawaii and the Marshall Islands is known as *Neanthes arenaceodentata* and has been the subject of considerable research in fields such as life history^1^, toxicology^2^, behavior^3^,^4^, vitellogensis^5^, feeding rate metabolism^6^, nervous system architecture^7^, proteomics^8^,^9^ and longevity^10^. Polychaetes exhibit variable reproductive patterns including pairing in mucoid tubes, external brooding and hermaphrodism^11^. Many polychaetes exhibit iteroparous reproduction, in which organisms reproduce multiple times. In nereidids, most females exhibit semelparous reproduction, in that they reproduce once and die^12^. The *N. acuminata* species complex is distinct from other nereidids in many ways. For example, they do not undergo sexual metamorphosis typical of many nereidids. Although females lay eggs and then die, males are capable of reproducing multiple times^1^. In contrast to most species of nereidids, males cannot be distinguished by the presence of sperm through the body wall. Sexes are determined by behavioral responses, in that individuals of the same sex will fight, whereas individuals of the opposite sex will lie side by side in a mucoid tube where ova are laid and fertilized^1^. A large mature worm of unknown sex is placed with a female with maturing ova visible through her body wall. The diploid numbers of chromosomes in this species complex are 2N = 18 (California), 2N = 22 (New England), and 2N = 28 (Hawaii)^3^, with 2N = 28 being most common to nereidids^3^. The male N. *arenaceodentata* undergoes behavioral, morphological and physiological changes during reproduction and is capable of reproducing nine times^10^. Their fighting pattern is altered during the incubation period, in that males fight any intruder. Sperm have never been observed (Reish, pers. obs). The male’s reproductive ability declines during the seventh reproductive period, after which the male is subsequently unable to fertilize ova.

Proteomic studies with *N. arenaceodentata* have analyzed the changes in the transition from egg to larvae^8^ and in males and females before and after fertilization^9^. Patterns of proteins and phosphoproteins were found to change as eggs become larvae^8^. Females, who die after fertilization, showed a 70% decrease in phosphoproteins. This study hypothesized that plasticity in protein expression patterns influences male reproductive success. The key questions were: (i) do males proceed through cycles of changes in protein expression patterns corresponding to successive reproductive periods? and (ii) are the changes in proteomic characteristics that influence male reproductive success achieved through repeated cycles of gametogenesis and sexual maturation? Male reproductive success rate was measured during each successive reproductive period. Males were found to gain body weight through six mating cycles. The number of males reproducing decreased after the fifth cycle and dropped significantly after the sixth.

## Results

### Variation in male reproductive success during reproductive periods

**Figure 1:**
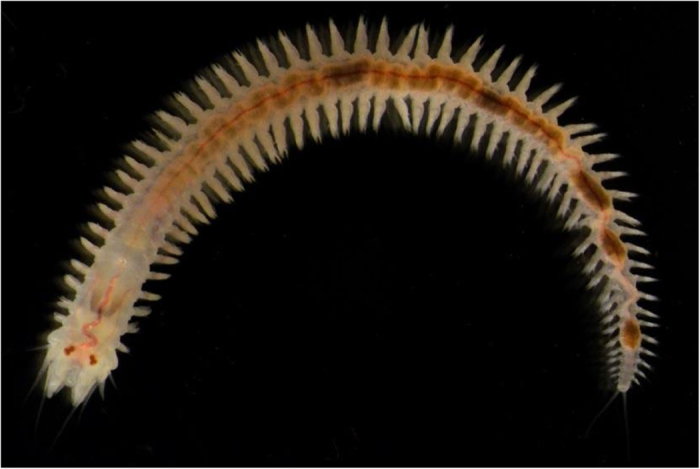
Male *Neanthes arenaceodentata*. Inbred population which was established in 1964 collected in Los Angeles Harbor, California, USA.

Table 1 summarizes the number of males during each reproductive period, their percent success in producing juveniles, the mean and standard deviation of the relative number of juveniles produced, and the length in days of each reproductive period. Reproductive success of male (Fig. 1), as measured by larvae present at the end of the reduced incubation period, was over 80% through the third period (1X–3X). This reproductive success rate started to decline from the fourth (70.8%) through the sixth (56.9%) reproductive period (4X–6X), accompanied by a decrease in the number of juveniles. The reproductive success rate and number of juveniles increased up to 67% through reproductive periods 7X and 8X. One male, approximately one year of age, reproduced successfully through nine periods (9X) but produced fewer than 50 juveniles.

### The proteome of male *Neanthes arenaceodentata*

**Figure 2:**
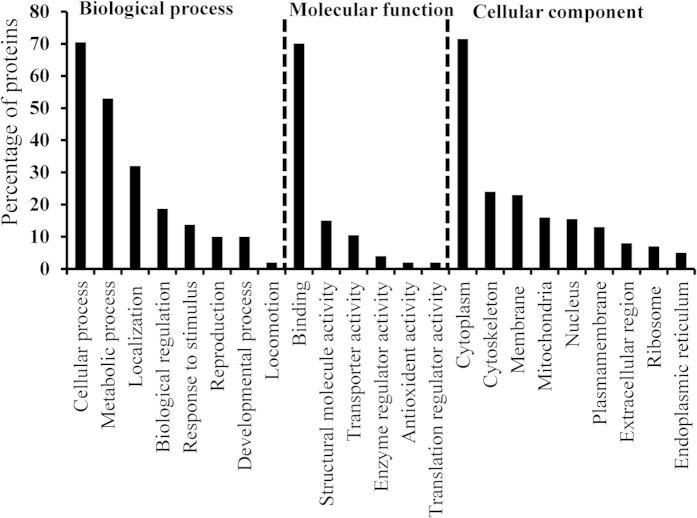
Gene ontology (GO) of all the proteins identified in male polychaetous annelid *Neanthes arenaceodentata*.

**Figure 3:**
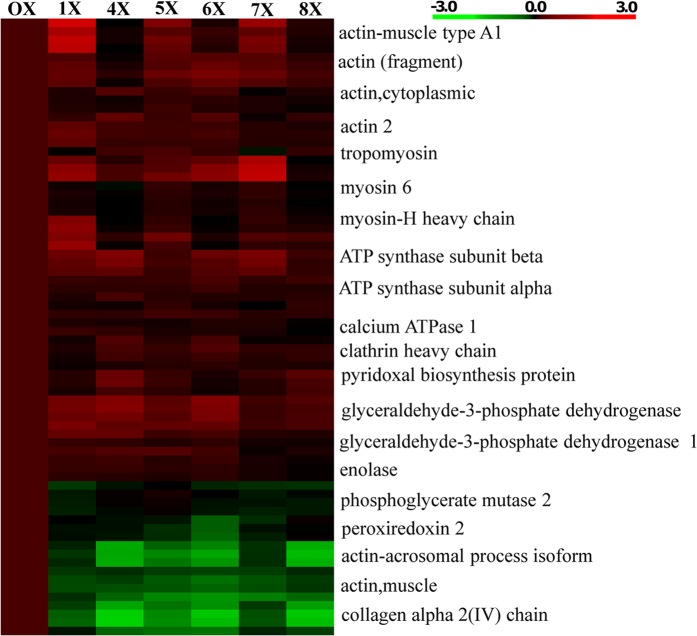
Heat map of differentially expressed proteins during reproductive periods in male polychaetous annelid *Neanthes arenaceodentata*. Red: up-regulation; green: down-regulation. The horizontal axis indicates the reproductive periods in the order 0X to 8X.

A total of 266 proteins were identified from 2250 spectra, of which 691 were unique. Proteins accounted for an average of 1.88 unique peptides, 2.59 unique spectra, 12.8 total spectra and 10.7% sequence coverage (Supplementary Table S1). Figure 2 lists the percentages of proteins belonging to each Gene Ontology (GO) term, such as biological process (BP), molecular function (MP), and cellular component (CC). The two most prominent BPs were cellular and metabolic processes, representing 70.5% and 53% of proteins, respectively. Ten percent of proteins were related to reproduction. Over 70% of proteins were associated with binding activity, followed by structural (15%) and transport (10.5%) activity of MP. Most proteins were localized to the cytoplasm, with 33% and 15.5% associated with the membrane and nucleus, respectively. Proteins were quantified relative to virgin males (0X), with 113 reporter ion intensities adjusted to 1. Twenty proteins showed changes in expression pattern through the first to eight reproductive periods (1X–8X). Fifteen of these proteins were up-regulated and 5 were down-regulated. Supplementary Table S2 shows the average fold changes and standard deviations of differentially expressed proteins obtained from three replicates. A heat map (Fig. 3) showed clusters of down-regulated (green) and up-regulated (red) proteins during successive reproductive stages (1X–8X), with the dark color representing reference values for 0X. Consistent changes were observed for the replicates.

### Changes in expression patterns of muscular and metabolic proteins

**Figure 4:**
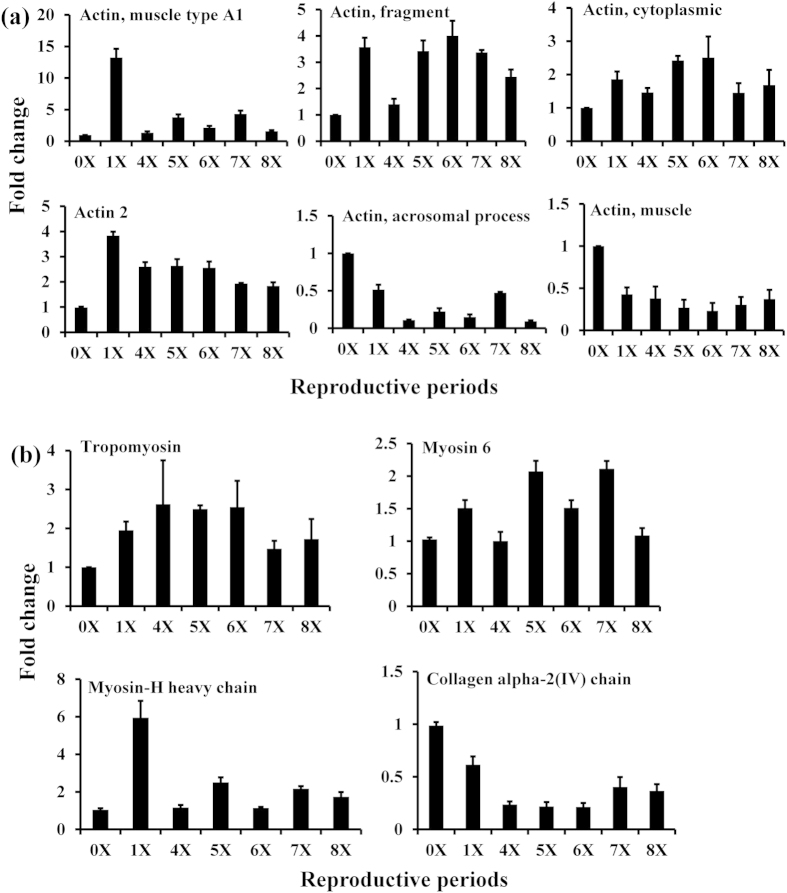
Differential expression of actins (**a**) and myosins (**b**) involved in muscular activity during reproductive periods in male polychaetous annelid *Neanthes arenaceodentata*.

**Figure 5:**
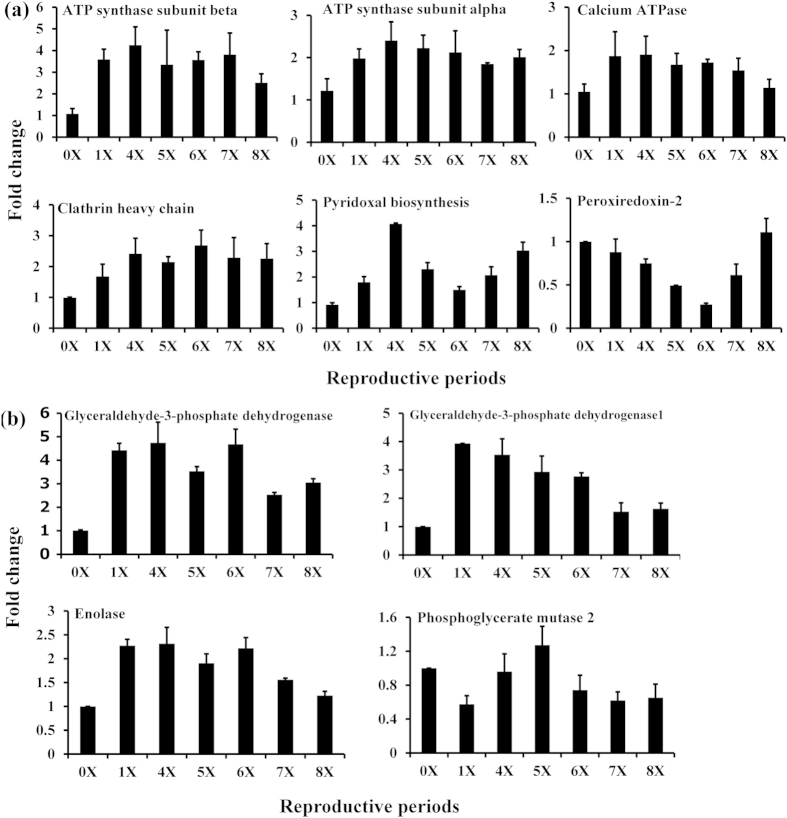
Differential expression of proteins of oxidative phosphorylation (**a**) and glycolysis (**b**) during reproductive periods in male polychaetous annelid *Neanthes arenaceodentata*.

Table 2 lists the differentially expressed proteins, along with a description of the number of peptides, spectra and sequence coverage matched to each protein. The expression of six actin family proteins, muscle-type A1, actin, actin (cytoplasmic), and actin-2, was increased from the first to sixth reproductive period (1X–6X), but decreased during periods 7X–8X (Fig. 4a). In contrast, actin-muscle and acrosomal process isoform expression decreased from 1X to 6X, but increased during 7X and 8X (Fig. 4a). The three muscle proteins, tropomyosin, myosin-6, and myosin heavy chain, increased, while collagen alpha-2 decreased, during all reproductive periods (1X–8X) (Fig. 4b). Expression of clathrin heavy chain, ATP synthase and calcium ATPase was up-regulated during all reproductive periods (Fig. 5a). Two antioxidant proteins, pyridoxal biosynthesis protein (PDX1) and peroxiredoxin-2 (PDX2), exhibited opposite expression patterns. PDX1 increased during all reproductive periods, whereas PDX2 expression was decreased from 1X to 6X, but increased thereafter (Fig. 5a). Glyceraldehyde-3-phosphate dehydrogenase (GAPDH) and enolase were dramatically up-regulated from 1X to 6X, but were reduced during 7X and 8X (Fig. 5b). The expression of phosphoglycerate mutase decreased during the first, sixth, seventh and eighth reproductive periods. Fifteen proteins involved in spermatogenesis, gonad development, oogenesis, and ovulation were identified. Table 3 lists proteins involved in reproductive success, along with a description of the number of peptides, spectra and sequence coverage. Several of these proteins, as marked by*, were differentially expressed during reproductive periods (1X–8X).

## Discussion

In this study, an iTRAQ-based quantitative proteomics approach was used to identify proteomic changes during successive reproductive periods in male *N. arenaceodentata*. Differentially expressed proteins involved in development, muscular activity, gametogenesis and sperm maturation were identified. N. *arenaceodentata* reproductive success was found to be associated with changes in protein expression patterns, with these proteins possibly influencing gametogenesis and sexual maturation.

### Body weight and reproductive success

Several proteins that influenced reproductive success of males were identified. The abundant expression of muscular and contractile proteins through the sixth reproductive period highlighted the importance of body weight and successful pairing for reproductive success^13^. Further, proteins associated with gametogenesis and sexual maturation may play a crucial role in determining the reproductive capacity of males^11^,^14^. The decrease in the number of larvae produced following the seventh reproductive period may be related to reductions in body weight, number of sperm and/or health. The higher reproductive rate, as shown by increased numbers of juveniles, may have resulted from the increase in body weight during the first six mating periods. Age was also found to negatively influence reproductive success rate, as shown by the decrease starting during the fifth mating period. Furthermore, only one male reached the ninth reproductive period, producing fewer than 50 juveniles.

### Complexity of worm proteome

Proteomics tools have been used to investigate protein expression dynamics during early development^8^, and to assess reproductive patterns in male and female *N. arenaceodentata*^9^. Previous studies are extended by proteomic changes in males occur through nine reproductive cycles. The extraction and purification of proteins from polychaetes poses a challenge in proteomic studies. For example, polychaetes are covered with self-secreted mucilage or slime^15^,^16^. Further, *Neanthes* adults are 2–4 cm in length and largely composed of cytoskeletal elements. Protein extraction from whole worms results in a high degree of complexity, which may prevent the identification of less abundant proteins by mass spectrometry. Proteomes degrade soon after lysis, requiring the addition of protease inhibitors during sample preparation^17^. Extraction of proteins was improved by homogenization, followed by precipitation with methanol and chloroform, enhancing protein recovery when compared with our previous study. Of the 266 proteins identified, 20 were differentially expressed and 15 were involved in reproductive success.

### Roles of muscular and contractile proteins

Increased expression of proteins associated with muscular and contractile properties during the first six reproductive periods resulted in an increase in worm body weight, suggesting a direct correlation between worm size. This finding would support the argument that the larger the worms; the higher the mating success, both in laboratory and field populations. Larger worms are generally more successful in fighting and pairing with other worms^18^. Several elastic or cytoskeletal proteins associated with body musculature, mechanical support and cellular signaling are present in polychaetes^19^,^20^,^21^. During the incubation period, the body of the male undulates to renew water^10^, and males fight off other individuals of both sexes to protect their embryos^1^. The abundant expression of non-muscle actins facilitates high speed contractions in mature males. Three connecting proteins have been identified in muscle tissue of *Neanthes* sp^22^. The contractile function of muscle tissue relies on the interactions between actins and myosins. Myosins synergistically modulate reproduction by interacting with other myosin isoforms and with actins^23^,^24^. These proteins showed varied expression patterns before and after spawning in *N*. *arenaceodentata*^8^,^9^. The up-regulation of several myosins and actins during reproduction may facilitate the increased body weight essential for muscular functions during mating suggesting that increased reproductive effort in males may be mediated by muscular and contractile proteins. Down-regulation of other actin proteins, including actin muscle and actin acrosomal process isoforms, after the seventh reproductive period may lead to reduced body weight decreasing reproductive effort. Sperm competition and production are reduced as males age.

### Proteins involved in sexual maturation and fertilization

Nereidids cease feeding at the end of maturation and depend on fat catabolism and muscle protein to fuel the growth of gametes^25^. In *Neanthes*, coelomic cells synthesize lipids required for the development of spermatogonia^26^. The abundant expression of glycolytic enzymes in males may indicate an increase in energy metabolism and related sexual maturation. GAPDH, enolase and phosphoglycerate mutase are enzymes involved in the process of sperm maturation^27^,^28^. GAPDH activity in sperm affects energy metabolism, maturation and fertilization, whereas the sperm-specific enolase isoform may play a major role during different stages of maturation. Differences in the glycosylation patterns of enolase-phosphatase E1 and 14-3-3 probably play important roles in polychaete male reproductive strategies^29^. ATP synthases display different expression profiles during sexual reproduction^30^. Abundant expression of ATP synthase in males may influence reproduction through oxidative phosphorylation. Increased mitochondrial ATPase activity in *N. virens* males may increase the reductive state of enzymes in the respiratory chain, facilitating the production of reactive oxygen species (ROS) and generating oxidative stress^31^. Altered expression of pyridoxal proteins in polychaetes may compensate for oxidative stress^32^,^33^.

The bodies of polychaetes are covered by a cuticle made of collagen fibers^34^. A differential distribution of collagen fibers has been reported in male *Teleostei* fish during spawning^35^. The numbers of collagen fibers are reduced due to degradation by proteinases. The clathrin protein complex plays an important role in oocyte maturation through spindle stabilization^36^. Other reproductive proteins, including 40S ribosomal protein S2, spectrin alpha chain, dynein heavy chain, nitric oxide synthase, and 26S protease regulatory subunit, are involved in oogenesis and ovulation^37^. T-complex protein 1 subunit and eukaryotic translation initiation factor facilitate the binding of sperm to the zona pellucida. No significant changes were observed in the expression patterns of these proteins, suggesting that their abundant synthesis was not required to achieve reproductive success.

In conclusion, the differentially expressed reproductive proteins identified in this study were found, for the first time, to be involved in sperm maturation and fertilization during successive reproductive periods. For example, ATP synthase subunit alpha, clathrin heavy chain and calcium-transporting ATPase were up-regulated, whereas actin, acrosomal process isoform and peroxiredoxin were down-regulated. Nevertheless, the expression of energy producing glycolytic proteins, such as GAPDH, enolase, and phosphoglycerate mutase, which also participate in sperm maturation, was unchanged. These findings suggest that the reproductive proteins identified in males are highly conserved and possibly have evolutionary implications in relation to sperm formation. However, such assumption warrants further investigations. *Neanthes* males exhibit different protein expression patterns which influence their reproductive capacity. Males capable of reproducing many times must proceed through cycles of protein expression during gametogenesis and sexual maturation. The majority of nereidid individuals of both sexes undergoes metamorphosis at maturity, spawn in the water column and die. It would be of interest to learn if both sexes undergo protein changes similar to those in male *N. arenaceodentata*.

## Materials and Methods

### Experimental worms

All worms were derived from the same inbred population, which was established from six animals collected in Los Angeles Harbor in 1964^1^. Those six specimens have undergone over 200 generations of reproduction without addition of new worms. Immature worms were taken from the laboratory colony of *N. arenaceodentata*. Juveniles were taken from the laboratory aquaria, placed in separate petri dishes and fed resoaked dried algae (*Enteromorpha* sp.) as needed. The sex of each worm was determined about one month later by the behavioral assay described above. Each male-female pair was placed in a one gallon (3.78 l) glass jar containing 750 ml of seawater, which was aerated, with the worms fed the green alga *Enteromorpha* sp. To insure that at least 10 males were obtained during each reproductive period, 140 male-female pairs were mated; however, 10 males were not obtained during the eighth and ninth reproductive periods (Table 1). The pairs were examined 3–5 times per week and their reproductive state was noted. The female was removed after the eggs were laid. The male incubation period lasts about 3–4 weeks, at which time the larvae leave the male’s tube and commence feeding. At the end of the first reproductive period, males are approximately 60 to 85 days old. However, since the male often eats the larvae, the young were removed at days 16 to 18. The number of larvae present was approximated numerically, with values of 1–4 indicating 1–50, 51–100, 101–200 and >200 larvae, respectively. A female with maturing ova was taken from the laboratory colony and placed with a male after each reproductive period. A minimum of five males from each reproductive period and five virgin males (Fig. 1) were frozen, except for periods 8 and 9 because fewer numbers reached that stage. Frozen males were shipped to King Abdullah University of Science and Technology, Saudi Arabia, for proteomic analysis.

### Proteome extraction, digestion and iTRAQ labeling

Two to three males from each reproductive period were suspended in lysis buffer containing 8 M urea and protease inhibitor. The lysates were homogenized (Wheaton Homogenizer, USA) and sonicated (Q Sonica, LLC, USA), and the contaminants were removed by precipitating lysates with methanol:chloroform (1:4, v/v). The pellets were vacuum dried and their protein concentrations measured using a 2-D Quant kit (GE Healthcare, UK). Protein (60 μg) pellets from each reproductive period were reduced, alkylated, and digested with trypsin (Promega, USA) at an enzyme:protein ratio of 1:40 for 16 h at 37 °C. The peptides were desalted using Sep-Pak C18 Vac cartridges (Water Corporation, USA) and labeled using iTRAQ Reagents-8plex Kit (Applied Biosystems, USA). The peptides were suspended in 27 μL of dissolution buffer, 50 μL isopropanol were added to each reagent and the solution transferred to the corresponding peptide samples. The reagents 113–114, 116–119 and 121 were labeled to 0X, 1X, 4X, 5X, 6X, 7X, and 8X, respectively depending on the reproductive period. Since only one male reached the ninth reproductive stage, this stage was not analyzed because the amount of tissue was insufficient. The reporter-peptide mixture was incubated for 60 min and all peptide samples labeled individually were pooled and dried. Samples were fractionated by cation exchange chromatography (SCX), with 15 peptide fractions desalted using Sep-Pak C18 Vac cartridges. The dried peptides were resuspended in 20 μL of LC sample buffer (97% H_2_0, 3% ACN, 0.1% formic acid) and desalted using C_18_ Zip-Tips (Millipore Ltd. USA). Six replicates of each sample were analyzed by mass spectrometry (LTQ-Orbitrap Velos; Thermo Scientific, Germany).

### Protein identification and quantitation

The MS spectra were searched against the SwissProt database (538,010 sequences) using the search engine Mascot v2.4.0 (Matrix Sciences Ltd, UK). The variable modifications were set to iTRAQ8plex (Y) and oxidation (M), and the fixed modifications to iTRAQ8plex (N-term), iTRAQ8plex (K), and Carbamidomethyl (C). Proteins were identified using Scaffold v4.3.2 (Proteome Software Inc. USA) software. Peptide and protein thresholds were set at 94% and 90%, respectively, a one peptide minimum, a 1.5% protein Prophet false discovery rate (FDR) and a 3% peptide Prophet FDR; and default Scaffold delta-mass correction. Results were quantitated using the Scaffold Q + algorithm^38^. The reference reporter 113 was normalized to produce a 1:1 fold change as described by Zhang *et al*.^39^. Replicates variations were evaluated by unsupervised multivariate principal component analysis (PCA), using the Multiple Array Viewer (MeV)^40^.

## Additional Information

**How to cite this article**: Chandramouli, K. H. *et al*. Proteomic Changes Associated with Successive Reproductive Periods in Male Polychaetous *Neanthes arenaceodentata*. *Sci. Rep*. **5**, 13561; doi: 10.1038/srep13561 (2015).

## Supplementary Material

Supplementary Information

## Figures and Tables

**Table 1:** Summary of reproductive success in *Neanthes arenaceodentata*.

Reproductive period	Number of pairs	Percent reproductive success	Relative number of juveniles*	Experimental days*
0X	5	—	—	—
1X	10	80%	2 ± 1.0	37.6 ± 14
2X	16	84.4	3.3 ± 0.8	65.4 ± 11
3X	19	80.7	2.6 ± 1.4	89.2 ± 15
4X	12	70.8	2.4 ± 1.2	148 ± 25
5X	18	62.2	2.2 ± 1.7	176.5 ± 35
6X	17	56.9	2.6 ± 1.1	201 ± 40
7X	13	67	2.5 ± 1.1	235 ± 32
8X	3	64.7	2.7 ± 1.4	260 ± 18
9X	1	100	1	273

^*^Mean and standard deviation.

**Table 2:** Proteins differentially expressed during successive reproductive periods in male polychaetous annelid *Neanthes arenaceodentata*.

Protein name	Expression during reproduction	Accession numbers	Molecular weight (KDa)	Identification probability (%)	Unique peptides	Unique spectrum	Total spectrum	Sequence coverage (%)
Actins								
Actin, muscle-type A1	up-regulated	ACT1_BOMMO	41.80	100	4	5	9	52.4
Actin (Fragment)	up-regulated	ACT_PROCL	36.00	99	1	2	3	20.2
Actin, cytoplasmic	up-regulated	ACTC_STYPL	42.00	98	1	2	4	43.2
Actin-2	up-regulated	ACT2_SACKO	42.00	99.7	2	2	7	51.9
Actin, muscle	down-regulated	ACTM_STRPU	41.00	99.9	1	1	3	33.4
Actin, acrosomal process isoform	down-regulated	ACTA_LIMPO	42.00	99.2	1	1	2	44.9
Muscle proteins								
Tropomyosin	up-regulated	TPM_HALDV	32.80	100	2	2	11	7.3
Myosin-6	up-regulated	MYH6_MOUSE	223.00	100	4	6	24	2.6
Myosin heavy chain, striated muscle	up-regulated	MYS_AEQIR	222.00	100	8	13	128	3.5
Collagen alpha-2(IV) chain	down-regulated	CO4A2_BOVIN	25.00	99.9	1	2	23	4.8
ATPase								
ATP synthase subunit alpha, mitochondrial	up-regulated	ATPA_CAEEL	57.70	100	2	2	14	15.2
ATP synthase subunit beta	up-regulated	ATPB_MAGSA	50.60	99	1	3	4	9.7
Sarcoplasmic/endoplasmic reticulum calcium ATPase 1	up-regulated	AT2A1_CHICK	109.00	100	4	7	13	7.9
Clathrin heavy chain	up-regulated	CLH_DROME	191.00	100	2	2	15	1.5
Antioxidants								
Pyridoxal biosynthesis protein (PDX1)	up-regulated	PDX1_GINBI	32.90	100.00%	4	6	15	10
Peroxiredoxin-2 (PDX2)	down-regulated	PRDX2_BOVIN	21.90	98.60%	1	1	7	5.5
Glycolysis								
Glyceraldehyde-3-phosphate dehydrogenase	up-regulated	G3P_BRUMA	36.00	99.9	1	2	6	4.1
Glyceraldehyde-3-phosphate dehydrogenase 1	up-regulated	G3P1_YEAST	35.70	98.1	1	4	11	2.4
Enolase	up-regulated	ENO_MASBA	48.00	99.2	1	1	2	3.1
2,3-bisphosphoglycerate-dependent phosphoglycerate mutase	down-regulated	GPMA_THETN	28.70	99.8	1	2	14	4.4

**Table 3:** Proteins involved in fertilization and spermatogenesis in male polychaetous annelid *Neanthes arenaceodentata*.

Proteins	Reproductive function	Accession number	Molecular weight (kDa)	Identification probability (%)	Unique peptides	Unique spectrum	Total spectrum
Actin, acrosomal process isoform*	spermatogenesis	ACTA_LIMPO	42	99.2	1	1	2
ATP synthase subunit alpha*	spermatid development	ATPA_DROME	59	100	11	17	62
40S ribosomal protein S2	oogenesis	RS2_DROME	29	100	3	3	5
Clathrin heavy chain*	sperm individualization	CLH_DROME	191	100	2	2	15
Spectrin alpha chain	oocyte construction	SPTCA_DROME	278	98.6	1	1	2
T-complex protein 1 subunit	binding of sperm to zona pellucida	TCPH_MOUSE	60	100	2	2	10
Peroxiredoxin-4*	spermatogenesis	PRDX4_HUMAN	31	99.9	2	2	9
Calcium-transporting ATPase 4*	spermatogenesis	AT2B4_RAT	133	99.4	1	2	5
Dynein heavy chain	ovarian fusome organization	DYHC_DROME	530	99.9	2	2	2
14-3-3 protein zeta	oocyte microtubule polarization	1433Z_DROME	28	99.2	1	1	1
Nitric oxide synthase	ovulation from ovarian follicle	NOS3_HUMAN	133	97.7	1	1	3
Eukaryotic translation initiation factor 5A	spermatogenesis	IF5A2_HUMAN	17	99.8	1	1	2
ATP synthase subunit beta*	gonad development	ATPB_CAEEL	58	99.7	1	2	3
T-complex protein 1 subunit beta	binding of sperm to zona pellucida	TCPB_BOVIN	57	99.8	1	1	2
26S protease regulatory subunit 4	sorocarp development	PRS4_DICDI	49	99.8	1	1	1

^*^differentially expressed proteins.
