# Supplementary material for: Proteomic Changes Associated with Successive Reproductive Periods in Male Polychaetous Neanthes arenaceodentata
Source: Sci Rep. 2015 Sep 4;5:13561. doi: 10.1038/srep13561 (PMC4559745; doi:10.1038/srep13561)
Supplement: Supplementary Information [file srep13561-s1.pdf]

Title: Proteomic Changes Associated with Successive Reproductive Periods in Male Polychaetous *Neanthes arenaceodentata*

Kondethimmanahalli H. Chandramouli[1], Donald Reish[2#], Huoming Zhang[3], Pei-Yuan Qian[4]* and Timothy Ravasi[1]*

Supplementary Table S1: Proteins identified in male polychaetous annelid *Neanthes arenaceodentata*

Protein name	Protein accession numbers	MW (Da)	identification probability	unique peptide count	unique spectrum count	spectrum count	seq coverage
ADP,ATP carrier protein, mitochondrial	ADT_ORYSJ	41,512.00	100.00%	3	5	28	6.81%
ATP synthase subunit alpha 2	ATPA2_DINSH	55,054.40	99.90%	1	2	19	13.10%
Tubulin alpha chain	TBA_EUGGR	49,892.90	99.80%	1	1	2	13.10%
Citrate synthase, mitochondrial	CISYM_DICDI	51,210.00	98.10%	1	1	2	2.61%
Adenylate kinase	KAD_XYLF2	19,943.00	95.80%	1	1	1	4.81%
HEAT repeat-containing protein 7B2	HTRB2_HUMAN	n/a	97.00%	1	1	2	0.00%
Glutamate synthase [NADH]	GLT1_YEAST	n/a	97.80%	1	1	1	0.47%
26S protease regulatory subunit 7	PRS7_XENLA	48,665.40	100.00%	3	3	15	8.31%
Malate dehydrogenase	MDH_SHEPW	31,882.40	99.20%	1	1	1	3.86%
Peptidyl-prolyl cis-trans isomerase 1	CYP1_SOYBN	18,178.50	99.60%	1	2	5	8.72%
Spectrin beta chain	SPTCB_DROME	n/a	99.30%	1	1	2	0.48%
60 kDa chaperonin	CH60_PSEFS	56,966.70	95.80%	1	1	1	4.01%
Pantothenate kinase	COAA_CORA7	35,178.10	95.30%	1	1	1	2.60%
Fructose-1,6-bisphosphatase class 1	F16PA_SULSY	35,376.40	99.70%	1	1	2	3.41%
Fructose-bisphosphate aldolase A	ALDOA_HUMAN	39,420.60	98.50%	1	1	1	4.12%
40S ribosomal protein SA	RSSA_PINFU	33,519.90	100.00%	3	3	10	8.31%
V-type proton ATPase catalytic subunit A	VATA_BOVIN,	68,345.30	100.00%	1	1	1	2.76%
Tubulin beta-4B chain	TBB4B_BOVIN,	49,830.70	100.00%	18	38	168	46.50%
Glutenin, high molecular weight subunit	GLT1_WHEAT	10,895.50	97.10%	1	1	6	10.90%

PC256							
40S ribosomal protein S5	RS5_BOVIN	22,877.00	100.00%	1	1	2	4.41%
Malate dehydrogenase, mitochondrial	MDHM_BOVIN	35,669.00	99.90%	1	1	2	3.25%
GTP-binding protein YPT31/YPT8	YPT31_YEAST	24,469.90	98.50%	1	1	1	4.48%
40S ribosomal protein S4	RS4_BOMMO	29,625.40	99.20%	1	1	1	2.66%
Actin-15A	ACT1_STRFN	41,827.90	96.50%	1	1	2	49.70%
Chaperone protein clpB 1	CLPB1_SYNPX	95,614.90	96.70%	1	1	1	1.74%
40S ribosomal protein S6	RS6_BOVIN	28,667.70	98.80%	1	1	3	3.21%
Keratin, type I cytoskeletal 10	K1C10_HUMAN	58,828.80	97.80%	1	1	1	1.54%
6-phosphofructokinase type C	K6PP_HUMAN	85,597.40	99.80%	1	1	8	1.15%
Glycocyamine kinase	KGCY_HEDDI	44,462.70	100.00%	8	15	165	20.40%
Actin, muscle 1A	ACT1_HALRO	42,045.90	99.50%	1	1	6	36.50%
Enolase	ENO_MASBA	48,007.40	99.20%	1	1	2	3.19%
T-complex protein 1 subunit eta	TCPH_MOUSE	59,653.20	100.00%	2	2	10	4.60%
Glycogen phosphorylase	PYGB_BOVIN	96,342.60	100.00%	5	7	13	5.46%
V-type ATP synthase beta chain	VATB_BORHD	48,007.20	95.10%	1	1	1	3.00%
Alpha-actinin, sarcomeric	ACTN_ANOGA	n/a	100.00%	4	5	13	5.31%
Actin-1 OS=Echinococcus granulosus	ACT1_ECHGR	41,829.00	99.90%	2	3	4	29.90%
Medium-chain specific acyl-CoA dehydrogenase	ACADM_PIG	46,486.50	99.70%	1	1	2	3.09%
Collagen alpha-2(IV) chain	CO4A2_BOVIN	25,060.80	99.90%	1	2	23	4.85%
Actin	ACT_CRYNV	41,737.60	99.80%	1	2	7	27.50%
1-deoxy-D-xylulose 5-phosphate reductoisomerase	DXR_PSEFS	42,367.60	95.60%	1	1	4	2.78%
Probable cytosol aminopeptidase	AMPA_PROM9	53,575.60	98.90%	1	1	2	2.45%
40S ribosomal protein S9	RS9_BOVIN	22,592.50	97.70%	1	1	2	4.64%
Tropomyosin	TPM_HALDV	32,824.20	100.00%	2	2	11	7.39%
Actin-1	ACT1_PHYIN	41,882.60	98.00%	1	1	2	25.50%
Actin	ACT_BRUMA	41,710.70	100.00%	3	3	44	57.70%
NADP-dependent malic enzyme	MAOX_PIG	62,008.50	97.50%	1	1	7	1.62%

Calcium-transporting ATPase	ATC_ARTSF	n/a	99.80%	1	1	2	9.97%
GTPase KRas	RASK_CYPCA	21,424.80	97.90%	1	1	2	6.38%
40S ribosomal protein S14a	RS14A_DROYA	16,264.90	99.90%	2	2	3	13.90%
40S ribosomal protein S18	RS18_BRABE	17,832.60	99.80%	1	1	3	5.92%
Isocitrate dehydrogenase	IDHP_BOVIN	50,739.90	100.00%	2	2	9	5.09%
Actin, cytoplasmic	ACTC_BIOOB	41,875.80	99.60%	1	1	1	51.60%
Serine hydroxymethyltransferase	GLYA_BORDL	45,437.70	95.00%	1	1	3	2.16%
Tubulin beta-1 chain	TBB1_CHICK	49,909.00	99.70%	1	2	5	42.90%
Hsp90 co-chaperone Cdc37-like 1	CD37L_PONAB	38,926.90	94.80%	1	1	2	2.67%
Heat shock cognate 70 kDa protein	HSP70_ONCMY	71,285.10	100.00%	6	6	24	11.10%
Actin	ACT_MESVI	41,590.20	99.90%	2	2	2	46.90%
78 kDa glucose-regulated protein	GRP78_XENLA	72,637.10	100.00%	3	3	8	10.30%
Peroxiredoxin-4.	PRDX4_CROAT	4,140.90	94.70%	1	1	1	50.00%
Creatine kinase M-type	KCRM_CHICK	43,328.90	99.90%	1	1	6	3.41%
Histone H3.1	H31_BOVIN	15,404.70	100.00%	3	3	6	15.40%
chromosomes protein 6	SMC6_DICDI	n/a	95.00%	1	1	2	0.68%
Myosin heavy chain	MYS_AEQIR	n/a	100.00%	8	13	128	3.51%
Serine/threonine-protein phosphatase 2A	2AAB_MOUSE	65,950.50	98.10%	1	1	4	0.00%
Prohibitin-2	PHB2_XENTR	33,461.80	99.90%	2	2	4	5.98%
Mechanosensory protein 2	MEC2_CAEEL	51,900.30	100.00%	4	6	15	5.82%
6-phosphofructokinase	K6PF_DROME	86,649.90	98.90%	1	1	6	1.14%
Light-independent protochlorophyllide reductase	BCHN_RHOCB	45,830.90	95.60%	1	1	1	1.89%
Stress-70 protein	GRP75_CRIGR	73,731.40	100.00%	2	2	3	3.39%
Betaine aldehyde dehydrogenase	BETB_OCHA4	52,219.00	97.20%	1	1	1	4.52%
ATP synthase subunit beta	ATPB_SYMTH	51,358.80	96.70%	1	1	3	7.20%
Keratin, type I cytoskeletal 9	K1C9_HUMAN	62,065.90	100.00%	3	4	9	8.03%
Janus kinase and microtubule-interacting protein 2	JKIP2_HUMAN	94,937.90	96.60%	1	1	1	0.99%

Voltage-dependent anion-selective channel protein 1	VDAC1_MOUSE	32,352.70	96.00%	1	2	2	2.70%
Intermediate filament protein A	IFEA_ASCSU	58,140.70	99.80%	1	2	8	2.21%
Glyceraldehyde-3-phosphate dehydrogenase	G3P_PHARH	36,082.90	99.80%	1	1	1	6.51%
Probable tyrosine-protein kinase kin-25	KIN25_CAEEL	n/a	97.90%	1	2	20	0.65%
Ras-related protein Rab-2	RAB2_LYMST	23,535.30	100.00%	3	3	4	20.80%
Actin-1	ACT1_ORYSI	41,813.80	94.80%	1	1	1	28.60%
Phosphoglycerate kinase	PGK_SCHMA	44,509.00	100.00%	1	1	20	3.61%
Actin-3	ACT3_DIPDE	41,912.80	99.80%	1	1	2	39.50%
NADH dehydrogenase	NDUS3_MOUSE	30,149.40	98.80%	1	1	4	4.94%
Proteasome subunit alpha	PSA6_BOVIN	27,399.50	99.80%	1	1	2	5.28%
GTP-binding nuclear protein Ran	RANT_MOUSE	24,452.00	96.40%	1	1	1	5.09%
6-phosphogluconate dehydrogenase	6PGD_SHEEP	52,972.20	99.80%	1	2	14	3.52%
ADP-ribosylation factor-like protein 8A	ARL8A_XENTR	21,404.80	97.20%	1	2	2	7.53%
Protein transport protein Sec61 subunit alpha isoform A	S61A1_ONCMY	52,201.20	99.00%	1	1	4	2.31%
DASH complex subunit dad1	DAD1_SCHPO	9,974.60	95.50%	1	1	1	10.60%
Actin, cytoskeletal 2	ACT2_LYTPI	41,874.90	98.20%	1	1	1	40.40%
Chaperone protein clpB	CLPB_CLOPE	97,944.30	96.70%	1	1	1	0.81%
Regulator of G-protein signaling 3	RGS3_HUMAN	n/a	95.50%	1	1	3	0.67%
ATP-binding cassette sub-family E member 1	ABCE1_HUMAN	67,316.40	99.70%	1	1	2	2.00%
Aspartate aminotransferase, cytoplasmic	AATC_MOUSE	46,232.30	99.20%	1	1	4	1.94%
Triosephosphate isomerase	TPIS_FUSNN	27,670.70	97.90%	1	1	1	5.98%
phosphoglycerate mutase	GPMA_THETN	28,753.30	99.80%	1	2	14	4.42%
40S ribosomal protein S26-A	RS26A_YEAST	13,505.00	96.50%	1	1	1	7.56%
Actin, cytoplasmic 2	ACTG_XENTR	41,765.80	100.00%	6	22	216	59.50%
Meprin A subunit alpha	MEP1A_RAT	85,141.50	96.90%	1	2	5	1.47%
ATP synthase subunit beta	ATPB_CAEEL	57,527.30	99.70%	1	2	3	14.30%

60S ribosomal protein L5	RL5_AEDAE	34,049.60	100.00%	2	2	11	7.74%
Actin-7	ACT7_ORYSI	41,615.40	98.80%	1	1	3	21.50%
Actin, muscle	ACTM_STRPU	41,557.20	99.90%	1	1	2	33.40%
Uncharacterized protein C2orf53	CB053_HUMAN	44,689.80	96.60%	1	1	2	2.67%
Tubulin alpha-1 chain	TBA1_DROME	49,908.40	100.00%	14	19	47	35.10%
Clathrin heavy chain	CLH_DROME	n/a	100.00%	2	2	9	1.55%
Triosephosphate isomerase	TPIS_CORDI	27,299.50	99.70%	1	2	14	5.77%
threonyl-tRNA synthetase 2	SYTC2_XENTR	93,330.80	94.50%	1	1	1	1.23%
Seryl-tRNA synthetase	SYS_ALHEH	47,871.40	96.60%	1	1	2	1.89%
Delta-1-pyrroline-5-carboxylate dehydrogenase	AL4A1_MOUSE	61,869.40	99.30%	1	1	6	1.60%
Adenosylhomocysteinase	SAHH_DROME	47,366.60	100.00%	3	3	14	6.71%
methylmalonyl-CoA mutase	MUTA_CAEEL	81,685.60	99.80%	1	1	2	2.15%
Short-chain specific acyl-CoA dehydrogenase	ACADS_BOVIN	44,553.90	98.10%	1	1	1	3.16%
Actin-3	ACT3_DICDI	41,842.00	99.90%	1	1	1	32.20%
Glial fibrillary acidic protein	GFAP_BOVIN	49,512.50	100.00%	3	3	11	3.97%
inorganic polyphosphate/ATP-NAD kinase	PPNK_VIBHB	32,382.00	97.00%	1	1	2	2.72%
ADP,ATP carrier protein 1	ADT1_YEAST	34,428.00	99.90%	2	4	5	3.56%
Phosphoglycerate kinase	PGK_CANGA	44,618.40	97.20%	1	2	5	1.92%
cAMP-dependent protein kinase regulatory subunit	KAPR_APLCA	42,737.80	98.90%	1	1	1	1.85%
Chaperone protein dnaK	DNAK_PAVLU	68,793.90	95.90%	1	1	2	2.70%
Myosin-4	MYH4_HUMAN	n/a	99.90%	1	2	3	2.58%
Protein phosphatase PP2A regulatory subunit A	2AAA_SCHPO	66,618.80	99.70%	1	1	12	2.03%
DNA ligase	DNLI_HAEIN	30,897.60	95.50%	1	1	1	3.73%
Actin-1	ACT1_SACKO	41,893.00	99.90%	1	1	7	46.00%
E3 ubiquitin-protein ligase BRE1A	BRE1A_CHICK	n/a	95.60%	1	1	1	0.81%
Guanine nucleotide-binding protein	GNAO_CAEEL	40,452.00	100.00%	2	4	15	6.21%

G(o) subunit alpha							
NADH-quinone oxidoreductase subunit I	NUOI_ACIBC	20,432.10	95.60%	1	1	1	5.56%
60S ribosomal protein L27	RL27_BOVIN	15,798.40	97.60%	1	1	1	5.88%
Enolase 1	ENO1_TOXGO	48,342.80	99.60%	1	1	1	4.50%
Betaine--homocysteine S-methyltransferase 1	BHMT1_XENTR	44,153.30	99.40%	1	1	2	3.97%
70 kDa neurofilament protein	NF70_LOLPE	71,004.20	98.70%	1	1	3	1.14%
Lethal(2) giant larvae protein homolog 2	L2GL2_HUMAN	n/a	99.30%	1	1	2	1.27%
aconitate hydratase	ACON_CAEEL	84,050.40	96.80%	1	1	2	1.03%
Paramyosin	MYSP_MYTGA	99,576.30	100.00%	5	6	30	3.70%
Propionyl-CoA carboxylase beta chain	PCCB_PIG	58,590.40	98.90%	1	1	3	2.60%
NAD(P) transhydrogenase	NNTM_BOVIN	n/a	96.40%	1	1	2	0.83%
tRNA pseudouridine synthase Pus10	PUS10_METTM	45,700.50	96.40%	1	1	7	1.99%
Histone H2B	H2B_LITVA,H2B_SIPNU	12,775.40	100.00%	8	11	35	57.80%
Endoplasmin	ENPL_BOVIN	92,429.40	99.70%	1	1	8	1.74%
Elongation factor 1-alpha	EF1A_ARTSA	50,568.20	100.00%	4	6	24	9.52%
delta-1-pyrroline-5-carboxylate synthetase	ALH13_CAEEL	86,529.20	100.00%	1	1	3	2.25%
Actin, cytoplasmic 1	ACTB_BOSMU	41,749.80	99.30%	1	1	1	42.90%
Eukaryotic translation initiation factor 5A-2	IF5A2_HUMAN	16,793.30	99.80%	1	1	2	7.84%
Tubulin alpha-1 chain	TBA1_PARLI	50,208.70	99.70%	1	2	19	33.20%
Serum basic protease inhibitor	IBPS_BOVIN	6,646.70	96.30%	1	1	2	20.00%
Tropomyosin A	TPM_ECHGR	32,269.40	99.20%	1	1	3	3.60%
Tubulin beta chain	TBB_PIG	49,860.90	94.90%	1	1	2	42.90%
ATP synthase subunit alpha	ATPA_NITHX	55,281.80	99.70%	1	2	7	11.00%
Actin (Fragment)	ACT_PROCL	36,125.80	99.00%	1	2	3	20.20%
ATP synthase subunit beta	ATPB_MAGSA	50,655.50	99.00%	1	3	4	9.70%
Actin	ACT_EXODE	41,707.10	97.20%	1	1	2	29.30%
60S ribosomal protein L18a	RL18A_DANRE	20,703.60	99.70%	1	1	4	7.39%

40S ribosomal protein S2	RS2_DROME	28,900.70	100.00%	3	3	5	9.74%
Nitric oxide synthase, endothelial	NOS3_HUMAN	n/a	97.70%	1	1	3	0.67%
Actin	ACT_FUCDI	41,794.30	98.30%	1	1	2	14.70%
Histone H4 type VIII	H48_CHICK	11,439.80	100.00%	7	15	83	58.30%
40S ribosomal protein S27a	RS27A_MANSE	9,301.20	100.00%	3	4	11	0.00%
Actin	ACT_ENCCU	41,994.20	98.80%	1	1	6	13.60%
Molybdenum import ATP-binding protein modC 2	MODC2_BRAJA	96,175.20	96.20%	1	1	1	0.89%
Actin-2	ACT2_LUMTE	41,840.70	100.00%	28	58	899	64.10%
Actin, cytoplasmic	ACTC_STYPL	41,930.70	98.00%	1	2	4	43.20%
ATP synthase subunit alpha	ATPA_KLULA	59,103.60	99.90%	1	2	5	13.50%
Actin-53 (Fragment)	ACT2_TOBAC	37,370.80	99.80%	2	2	2	27.10%
6-phosphogluconate dehydrogenase	6PGD_MOUSE	53,248.80	99.70%	1	1	8	4.35%
Keratin, type II cytoskeletal 2 epidermal	K22E_HUMAN	65,433.90	99.70%	1	1	3	3.29%
60S ribosomal protein L13	RL13_SCHMA	21,294.40	97.10%	1	1	4	4.89%
Peroxiredoxin-2	PRDX2_BOVIN	21,945.90	98.60%	1	1	7	5.53%
Actin-2	ACT2_SACKO	41,883.90	99.70%	2	2	7	51.90%
Keratin, type II cytoskeletal 1	K2C1_HUMAN	66,040.30	100.00%	5	5	8	8.39%
Sodium/potassium-transporting ATPase subunit alpha	ATNA_DROME	n/a	100.00%	3	3	6	11.20%
Sodium/potassium-transporting ATPase subunit alpha-3	AT1A3_HUMAN	n/a	100.00%	11	16	41	12.00%
Succinyl-CoA ligase [GDP-forming] subunit beta	SUCB2_BOVIN	46,692.10	99.70%	1	1	2	2.31%
Delta-aminolevulinic acid dehydratase	HEM2_MOUSE	36,023.70	98.00%	1	2	5	3.03%
Uncharacterized protein ZMO1242	Y1242_ZYMMO	43,500.20	97.50%	1	1	3	2.63%
Histone H2A.V	H2AV_BOVIN	13,509.20	97.90%	1	1	1	20.30%
Actin	ACT_YARLI	41,800.50	99.40%	1	1	2	23.70%
ATP synthase subunit beta	ATPB_ALHEH	49,574.90	99.70%	1	2	3	4.15%
2-oxoglutarate dehydrogenase	OGDHL_PONAB	n/a	97.90%	1	1	1	0.79%
Fructose-bisphosphate aldolase 2	ALF2_CAEEL	38,847.00	99.90%	2	2	3	7.10%

Electron transfer flavoprotein subunit beta	ETFB_BOVIN	27,763.20	96.60%	1	1	4	3.53%
Actin-2	ACT2_PEA	41,768.80	99.50%	1	2	16	25.30%
Triosephosphate isomerase	TPIS_CULTA	26,389.10	100.00%	2	3	21	6.48%
Tubulin alpha-2 chain	TBA2_DROME	49,966.50	99.60%	1	1	3	26.70%
Dihydrolipoyl dehydrogenase	DLDH_CANFA	54,153.20	94.70%	1	1	1	2.16%
40S ribosomal protein S16	RS16_SPOFR	17,010.20	99.90%	2	2	5	5.96%
Aspartate aminotransferase	AAT2_ARATH	44,267.20	98.10%	1	1	2	1.98%
Peroxiredoxin-4	PRDX4_HUMAN	30,541.10	99.90%	2	2	9	6.64%
Tubulin beta-1 chain	TBB1_BRUPA	50,191.40	99.10%	1	2	4	17.20%
Succinate dehydrogenase flavoprotein subunit	DHSA_ORYSJ	68,854.60	97.90%	1	1	2	1.75%
citrate synthase 1	CISY1_AEDAE	51,641.60	100.00%	2	2	7	5.14%
14-3-3-like protein 2	14332_CAEEL	28,068.50	100.00%	6	6	8	21.00%
Alpha-enolase	ENOA_ALLMI	47,323.30	100.00%	2	3	6	7.60%
1-Cys peroxiredoxin	1CPX_DIRIM	26,343.30	99.70%	1	1	7	4.68%
Terpenoid synthase 1	TPS01_ARATH	69,622.30	95.80%	1	1	1	1.17%
Mitochondrial-processing peptidase subunit beta	MPPB_HUMAN	54,367.10	100.00%	2	2	5	3.68%
ATP synthase subunit alpha	ATPA_CAEEL	57,788.50	100.00%	2	2	14	15.20%
ATP synthase subunit alpha	ATPA_DROME	59,423.30	100.00%	11	17	62	20.30%
T-complex protein 1 subunit beta	TCPB_BOVIN	57,476.70	99.80%	1	1	2	2.24%
Succinate dehydrogenase flavoprotein subunit B	DHSAB_XENLA	72,754.70	99.90%	1	1	8	1.65%
Actin-2	ACT2_ONCVO	41,819.70	99.20%	1	1	1	57.70%
Transitional endoplasmic reticulum ATPase	TERA_BOVIN	89,332.70	99.60%	1	1	2	1.24%
ATP synthase subunit beta	ATPB_BARBK	57,303.00	99.70%	1	1	24	8.01%
Sarcoplasmic/endoplasmic reticulum calcium ATPase 1	AT2A1_CHICK	n/a	100.00%	4	7	13	7.95%
Ras-related protein Rap-1A	RAP1A_BOVIN	20,987.30	99.30%	1	1	2	5.98%

40S ribosomal protein S20	RS20_BOVIN	13,373.00	97.80%	1	1	7	10.10%
Probable 26S protease regulatory subunit S10B	PRS10_CAEEL	45,860.60	99.80%	1	1	1	3.94%
Cartilage oligomeric matrix protein	COMP_RAT	82,661.20	99.00%	1	2	3	1.32%
Myosin-7B	MYH7B_MOUSE	n/a	99.80%	1	3	6	1.24%
DNA ligase	DNLJ_PROM1	77,846.50	95.60%	1	1	1	1.16%
ATP synthase subunit beta	ATPB_HEMPU	56,075.40	95.10%	1	1	1	17.60%
Malate dehydrogenase	MDH_HAEIN	32,542.80	99.70%	1	2	2	3.86%
AP-2 complex subunit alpha-2	AP2A2_BOVIN	n/a	99.20%	1	1	2	0.96%
V-type proton ATPase subunit D	VATD_MANSE	27,506.80	99.70%	1	1	8	4.47%
Actin-3	ACT3_SOYBN	41,608.90	100.00%	1	1	2	14.40%
RNA polymerase II transcription subunit 13	SSN2_YARLI	n/a	97.30%	1	1	19	0.68%
Adenylate kinase	KAD_WOLSU	20,843.50	98.40%	1	1	1	4.74%
Plasma membrane calcium-transporting ATPase 4	AT2B4_RAT	n/a	99.40%	1	2	5	0.91%
adenosylhomocysteinase 2	SAHH2_HUMAN	58,951.50	99.80%	1	1	6	2.26%
ATP synthase subunit beta	ATPB_BRUA2	54,791.20	99.60%	1	1	1	15.90%
Virion host shutoff protein	VHS_SUHVK	40,910.60	96.60%	1	1	1	3.29%
Porphobilinogen deaminase	HEM3_ERWCH	9,922.70	95.30%	1	1	1	12.40%
Acyl-coenzyme A synthetase ACSM1	ACSM1_BOVIN	64,923.70	96.60%	1	1	1	1.56%
Myosin-6	MYH6_MOUSE	n/a	100.00%	4	6	24	2.68%
S-adenosylmethionine synthetase	METK_PELTS	43,621.70	98.90%	1	1	3	3.79%
Elongation factor 2	EF2_DROME	94,460.70	100.00%	1	1	4	1.07%
14-3-3 protein zeta	1433Z_DROME	28,228.30	99.20%	1	1	1	13.30%
Tubulin beta-4 chain	TBB4_CAEEL	49,799.80	99.90%	2	2	4	27.70%
Ras-related protein Rab-1A	RAB1A_CANFA	22,678.50	100.00%	2	2	5	10.70%
60S ribosomal protein L4-A	RL4A_XENLA	44,936.70	99.50%	1	1	2	2.78%
Calcium-transporting ATPase	ATC1_ANOGA	n/a	100.00%	8	11	30	8.45%
Guanine nucleotide-binding protein subunit beta	GBB_LOLFO	37,323.50	100.00%	4	5	12	13.20%

Glyceraldehyde-3-phosphate dehydrogenase	G3P_BRUMA	36,155.40	99.90%	1	2	6	4.13%
Spectrin alpha chain	SPTCA_DROME	n/a	98.60%	1	1	2	0.33%
Protein traI	TRAI1_ECOLI	n/a	96.10%	1	1	1	0.74%
Malate dehydrogenase	MDHC_BOVIN	36,483.90	97.90%	1	1	1	2.99%
40S ribosomal protein S3	RS3_BOVIN	26,688.60	100.00%	4	5	21	19.30%
phosphoglucomutase	PGM_SCHPO	60,600.60	100.00%	2	2	6	3.97%
Trypsin	TRYP_PIG	24,409.30	100.00%	5	11	102	32.90%
Proteasome subunit beta type-5	PSB5_HUMAN	28,481.00	95.40%	1	1	1	3.04%
Heat shock protein HSP 90-alpha	HS90A_BOVIN	84,734.20	100.00%	3	3	12	5.05%
Glyceraldehyde-3-phosphate dehydrogenase	G3P_CANAW	35,833.60	98.40%	1	1	1	8.06%
Beta-actin-like protein 2	ACTBL_MOUSE	42,005.10	99.90%	1	1	15	19.90%
40S ribosomal protein S25	RS25_BRABE	13,677.00	97.20%	1	1	1	8.13%
Actin, cytoplasmic 1	ACTB_ORYLA	41,739.90	99.50%	1	1	1	48.00%
Prohibitin	PHB2_DICDI	29,804.60	99.10%	1	1	2	3.31%
Glyceraldehyde 3-phosphate-dehydrogenase	G3P_NEUCR	36,292.60	99.90%	1	2	8	2.37%
Histone H2A type 1-A	H2A1A_HUMAN	14,234.20	100.00%	3	3	5	14.50%
ATP synthase subunit beta	ATPB_DROME	54,109.00	100.00%	7	15	80	17.00%
Tubulin beta chain	TBB_NEUCR	49,867.50	100.00%	2	3	10	9.84%
isoleucyl-tRNA synthetase	SYIC_ENCCU	n/a	95.60%	1	1	1	0.79%
Actin, muscle-type A1	ACT1_BOMMO	41,877.10	100.00%	4	5	9	52.40%
Glyceraldehyde-3-phosphate dehydrogenase 1	G3P1_YEAST	35,750.20	98.10%	1	4	9	2.41%
Myoferlin	MYOF_HUMAN	n/a	99.20%	1	1	2	0.53%
Dynein heavy chain	DYHC_DROME	n/a	99.90%	2	2	2	0.35%
Creatine kinase M-type	KCRM_BOVIN	42,989.50	100.00%	2	3	9	5.77%
26S protease regulatory subunit 4	PRS4_DICDI	49,193.20	99.80%	1	1	1	2.73%
Actin, cytoskeletal 4 (Fragment)	ACT4_LYTPI	17,150.20	100.00%	2	2	6	35.10%
pyridoxal biosynthesis protein PDX1	PDX1_GINBI	32,944.30	100.00%	4	6	15	10.00%

F-box only protein 16	FBX16_MOUSE	38,988.60	94.50%	1	1	2	2.40%
Propionyl-CoA carboxylase alpha chain	PCCA_CAEEL	79,761.90	98.90%	1	1	4	1.38%
Small COPII coat GTPase sar-1	SAR1_NEUCR	21,583.50	98.50%	1	1	2	6.88%
Transmembrane emp24 domain	TMEDE_DROER	25,183.60	99.70%	1	1	3	7.87%
Cytochrome c oxidase subunit 2	COX2_ALOPA	26,510.50	96.40%	1	1	2	3.03%
Dihydrolipoyllysine-residue succinyltransferase	ODO2_HUMAN	48,729.20	99.00%	1	1	1	1.77%
Pancreatic trypsin inhibitor	BPT1_BOVIN	10,903.10	99.80%	1	1	9	13.00%
Ryanodine receptor 3	RYR3_HUMAN	n/a	99.80%	1	1	2	0.23%
Eukaryotic initiation factor 4A-II	IF4A2_BOVIN	46,403.60	100.00%	2	2	5	8.60%
**Total: 266 proteins**				**1.883**	**2.598**	**12.846**	**10.71%**

Supplementary Table S2: Fold change values derived from replicates of differentially expressed proteins during reproductive period. Average fold change and SD for each protein is shown in Fig 2 and 3.

	Successive reproductive periods and corresponding iTRAQ values							Replicates
	0X	1X	4X	5X	6X	7X	8X	
**Differentially expressed proteins**	113	114	116	117	118	119	121	
Actin, muscle type A1	N/A	19.8392	1.0634	5.0009	1.6646	N/A	1.8731	R1
	0.5753		1.461	3.3533	2.435	3.7527	1.7715	R2
	0.3242	14.2173	1.4848	4.2677	2.1068	4.7843	1.546	R3
	0.289	12.2598	1.0701	3.7889	1.8621	4.4617	1.4608	R4
**AVG**	**0.396166667**	**13.23855**	**1.3386333**	**3.8033**	**2.134633**	**4.3329**	**1.592767**	
SD	0.127478948	0.97875	0.1901302	0.373441	0.234712	0.430884	0.131083	
Actin,fragment	1	3.0438	1.2753	3.4417	3.3731	3.3768	2.0768	R1
	1	3.7424	1.6431	3.1638	4.0865	3.4664	2.452	R2
	1	3.785	N/A	3.9856	4.737	N/A	2.7577	R3
	1	3.7257	1.2926	3.1091	3.8175	3.2815	2.4878	R4
**AVG**	**1**	**3.574225**	**1.4036667**	**3.42505**	**4.003525**	**3.3749**	**2.443575**	
SD	0	0.307003	0.1694522	0.347336	0.494194	0.075497	0.2425	
Actin, cytoplasmic	1	1.7432	N/A	2.3307	2.8259	1.1461	1.832	R1
	1	1.7041	1.372	2.3042	2.0801	1.7048	1.4327	R2
	1	1.7908	1.5577	2.6045	1.8898	1.69	1.2334	R3
	1	2.2055	N/A	2.4585	3.2422	1.2873	2.2575	R4
**AVG**	**1**	**1.8609**	**1.46485**	**2.424475**	**2.5095**	**1.45705**	**1.6889**	
SD	0	0.20131	0.09285	0.119192	0.548947	0.245536	0.392716	
Actin 2	1	3.7639	2.6722	2.6212	2.5414	1.9514	1.7188	R1
	1	4.0133	2.6913	2.6325	2.8953	1.9459	1.9986	R2

	0.9342	3.7186	2.3293	2.3218	2.5099	1.9131	1.7996	R3
	1	N/A	2.7166	2.9717	2.3004	N/A	N/A	R4
**AVG**	**0.98355**	**3.831933**	**2.60235**	**2.6368**	**2.56175**	**1.9368**	**1.839**	
SD	0.028492236	0.129572	0.1584302	0.230024	0.213695	0.016908	0.117576	
Actin, acrosomal process isoform	1	0.57	0.11	0.2699	0.187	0.4599	0.1044	R1
	1	0.4416	0.1159	0.1828	0.1195	0.4793	0.0794	R2
	1	0.535	0.1073	0.2222	0.1464	0.4833	0.095	R3
**AVG**	**1**	**0.515533**	**0.1110667**	**0.224967**	**0.150967**	**0.474167**	**0.092933**	
SD	0	0.066377	0.0043981	0.043616	0.033981	0.012516	0.012627	
Actin,muscle	1	0.4225	0.4904	0.3978	0.3742	0.3972	0.5179	R1
	1	0.3502	0.3892	0.2933	0.2327	0.3138	0.4093	R2
	1	0.3419	0.3269	0.3042	0.2585	0.3361	0.4399	R3
	1	0.4797	0.262	0.1849	0.1515	0.2069	0.257	R4
	1	N/A	0.592	N/A	N/A	0.391	0.3734	R5
	1	0.4202	0.2228	0.1679	0.1489	0.1849	0.2364	R6
**AVG**	**1**	**0.4029**	**0.38055**	**0.26962**	**0.23316**	**0.304983**	**0.372317**	
SD	0	0.057184	0.1404781	0.094487	0.092605	0.090542	0.108538	
Tropomyosin								
	0.9355	5.9698	2.8345	3.3568	5.6107	12.7408	1.4474	R1
	1	6.682	2.9343	4.4748	5.8382	14.448	1.6678	R2
**AVG**	**0.96775**	**6.3259**	**2.8844**	**3.9158**	**5.72445**	**13.5944**	**1.5576**	
SD	0.045608387	0.503601	0.0705693	0.790545	0.160867	1.207173	0.155846	
Myosin 6	1	1.3618	0.8012	2.3115	1.5335	2.1908	0.9672	R1
	1	1.6363	1.1399	2.0417	1.6666	2.2073	1.1814	R2
	1.0151	1.4327	1.0636	1.9595	1.3859	1.9294	0.9894	R3

	1.0741	1.5835	0.9844	1.9589	1.4397	2.0988	1.1901	R4
**AVG**	**1.0223**	**1.503575**	**0.997275**	**2.0679**	**1.506425**	**2.106575**	**1.082025**	
SD	0.030535471	0.110823	0.1258492	0.144619	0.106498	0.11033	0.104067	
Myosin-H heavy chain	1	5.1168	1.0005	2.2388	1.0805	2.2691	1.4652	R1
	1	5.8429	1.2459	2.4922	1.1982	2.0188	1.7692	R2
	1.1361	6.9015	1.2401	2.7778	1.1399	2.2147	1.9689	R3
**AVG**	**1.045366667**	**5.953733**	**1.1621667**	**2.502933**	**1.139533**	**2.167533**	**1.734433**	
SD	0.064158155	0.732804	0.1143401	0.220177	0.048052	0.10749	0.207099	
Collagen alpha-2(IV) chain	1	0.6958	0.2596	0.2419	0.2294	0.5592	0.3824	R1
	1	0.6007	0.2238	0.2368	0.2019	0.3874	0.3126	R2
	1	0.5145	0.2006	0.1857	0.208	0.3311	0.3236	R3
	0.9111	0.652	0.2622	0.2822	0.2653	0.4938	0.4493	R4
**AVG**	**0.977775**	**0.61575**	**0.23655**	**0.23665**	**0.22615**	**0.442875**	**0.366975**	
SD	0.04445	0.077889	0.0296883	0.039574	0.02864	0.102788	0.062858	
ATP synthase subunit beta*	1	4.1507	5.2169	2.5564	4.0903	4.6955	2.6179	R1
	1	3.6961	4.6855	2.7379	3.3461	4.3878	2.1605	R2
	0.8782	3.5064	3.6827	2.3808	3.2456	3.7291	2.2227	R3
	1.4322	3.0031	3.419	5.7254	3.5778	2.4636	3.0488	R4
**AVG**	**1.0776**	**3.589075**	**4.251025**	**3.350125**	**3.56495**	**3.819**	**2.512475**	
SD	0.243272851	0.475072	0.843987	1.590214	0.376845	0.98944	0.41094	
ATP synthase subunit alpha*	1.1121	2.0618	2.178	2.0573	2.5988	1.8584	1.774	R1
	1.5376	1.7356	2.9123	1.9786	1.8428	1.8207	1.9754	R2
	1	N/A	N/A	2.2233	1.5509	N/A	2.1825	R3
		2.1553	2.1247	2.6576	2.5037	1.8728	2.1178	R4
**AVG**	**1.216566667**	**1.984233**	**2.405**	**2.2292**	**2.12405**	**1.850633**	**2.012425**	

SD	0.283616649	0.220339	0.4401422	0.303266	0.508961	0.026904	0.180967	
calcium ATPase 1	0.9049	1.6766	1.609	1.4466	1.7781	1.8013	1.136	R1
	1	2.5061	2.2088	1.6222	1.6748	1.5857	0.9548	R2
	1.2454	1.4372	N/A	1.9591	N/A	1.2473	1.338	R3
**AVG**	**1.0501**	**1.8733**	**1.9089**	**1.675967**	**1.72645**	**1.544767**	**1.142933**	
SD	0.17569169	0.560941	0.4241226	0.260446	0.073044	0.279259	0.191694	
Clathrin*	1	1.7628	2.83	2.3541	3.1446	2.2228	2.1499	R1
	1	1.4726	2.6667	2.1302	2.5452	1.8864	2.2096	R2
	0.9728	1.2899	1.6965	1.9399	2.0592	1.8117	1.761	R3
		2.1983	2.4762	2.1747	3.0051	3.2286	2.9194	R4
**AVG**	**0.990933333**	**1.6809**	**2.41735**	**2.149725**	**2.688525**	**2.287375**	**2.259975**	
SD	0.015703927	0.396098	0.5018444	0.170099	0.491534	0.652463	0.482518	
Pyridoxal biosynthesis protein (PDX1)	1	1.9446	4.0526	2.4211	1.6349	2.4475	3.4012	R1
	0.8411	1.8994	4.0964	2.4883	1.4757	1.9161	2.9154	R2
	0.9102	1.5557	N/A	2.0262	1.3888	1.8575	2.7917	R3
**AVG**	**0.9171**	**1.7999**	**4.0745**	**2.311867**	**1.4998**	**2.0737**	**3.0361**	
SD	0.0796744	0.212688	0.0309713	0.249666	0.124807	0.325044	0.322178	
Peroxiredoxin-2 (PRDX2)*	1	1.0514	0.7732	N/A	0.282	0.4961	1.2535	R1
	1	0.7803	0.783	0.4937	0.2846	0.7456	0.9426	R2
	1	0.8082	0.6911	0.4948	0.2588	0.604	1.1337	R3
**AVG**	**1**	**0.879967**	**0.7491**	**0.49425**	**0.275133**	**0.615233**	**1.109933**	
SD	0	0.14912	0.0504679	0.000778	0.014205	0.125129	0.156807	
Glyceraldehyde-3-phosphate dehydrogenase	1	4.3479	5.3026	3.3847	5.3415	2.6734	3.2752	R1

	1	4.5106	5.6175	3.5896	4.8758	2.485	2.8827	R2
	1	4.0711	4.3355	3.3714	4.7065	2.4633	3.026	R3
	1.0527	4.7786	3.7269	3.7825	3.8128	2.5182	3.032	R4
**AVG**	**1.013175**	**4.42705**	**4.745625**	**3.53205**	**4.68415**	**2.534975**	**3.053975**	
SD	0.02635	0.296386	0.8710894	0.194557	0.639945	0.095005	0.16283	
Glyceraldehyde-3-phosphate dehydrogenase 1	1	3.9347	3.9318	2.5461	2.863	1.866	1.7695	R1
	1	N/A	N/A	N/A	N/A	1.4639	1.4068	R2
		N/A	3.1416	3.3285	2.6814	1.2595	1.719	R3
**AVG**	**1**	**3.9347**	**3.5367**	**2.9373**	**2.7722**	**1.5298**	**1.631767**	
SD	0	0	0.5587558	0.55324	0.128411	0.308574	0.196456	
Enolase	1	2.3415	2.6072	1.9161	2.4755	1.5967	1.3228	R1
	1	2.359	2.4001	2.0974	2.1234	1.5519	1.1519	R2
	1	2.1209	1.9389	1.7065	2.0605	1.531	1.2045	R3
**AVG**	**1**	**2.2738**	**2.3154**	**1.906667**	**2.2198**	**1.559867**	**1.2264**	
SD	0	0.132704	0.3421064	0.195621	0.223665	0.033567	0.087529	
phosphoglycerate mutase 2	1	0.4287	0.7152	1.0013	0.5387	0.4929	0.4593	R1
	1	0.6513	1.1465	1.5317	0.9646	0.6717	0.8513	R2
	1	0.6296	1.1209	1.3306	0.7565	0.7244	0.65	R3
	1	0.5893	0.8584	1.2289	0.7094	0.5922	0.6492	R4
**AVG**	**1**	**0.574725**	**0.96025**	**1.273125**	**0.7423**	**0.6203**	**0.65245**	
SD	0	0.100682	0.2089022	0.220612	0.175266	0.100828	0.160067	
